# Insulin-like Growth Factor-2 (IGF-2) in Fibrosis

**DOI:** 10.3390/biom12111557

**Published:** 2022-10-25

**Authors:** Yuhan Zhu, Lin Chen, Binyu Song, Zhiwei Cui, Guo Chen, Zhou Yu, Baoqiang Song

**Affiliations:** Department of Plastic and Reconstructive Surgery, Xijing Hospital, Fourth Military Medical University, Xi’an 710032, China

**Keywords:** insulin-like growth factor 2 (IGF-2), extracellular matrix (ECM), fibrosis, IGF-1R, IR

## Abstract

The insulin family consists of insulin, insulin-like growth factor 1 (IGF-1), insulin-like growth factor 2 (IGF-2), their receptors (IR, IGF-1R and IGF-2R), and their binding proteins. All three ligands are involved in cell proliferation, apoptosis, protein synthesis and metabolism due to their homologous sequences and structural similarities. Insulin-like growth factor 2, a member of the insulin family, plays an important role in embryonic development, metabolic disorders, and tumorigenesis by combining with three receptors with different degrees of affinity. The main pathological feature of various fibrotic diseases is the excessive deposition of extracellular matrix (ECM) after tissue and organ damage, which eventually results in organic dysfunction because scar formation replaces tissue parenchyma. As a mitogenic factor, IGF-2 is overexpressed in many fibrotic diseases. It can promote the proliferation of fibroblasts significantly, as well as the production of ECM in a time- and dose-dependent manner. This review aims to describe the expression changes and fibrosis-promoting effects of IGF-2 in the skin, oral cavity, heart, lung, liver, and kidney fibrotic tissues.

## 1. Introduction

Fibrosis has been a difficult challenge for the world to overcome. This pathological feature is present in a wide range of diseases and has a dramatic impact on tissue and organ function, leading to organ failure and even death, in severe cases [1]. IGF-2, a member of the insulin family, is an anabolic factor that promotes cell proliferation and migration [2]. In recent years, high IGF-2 expression has been found in various fibrotic diseases such as liver cirrhosis, idiopathic pulmonary fibrosis, and hypertrophic scars [3,4,5]. In addition, IGF-2 has also been studied for its role in creating a fibrotic environment both inside and outside cells, thereby promoting the development and progression of fibrotic diseases, confirming that IGF-2 plays an important role in tissue and organ fibrosis [6,7]. No researchers have yet summarized the relevance of IGF-2 to fibrotic diseases. We hope that this review will deepen researchers’ understanding of IGF-2-promoting fibrotic diseases and provide new ideas for the treatment of fibrotic diseases.

## 2. IGF-2

### 2.1. IGF-2 Gene and Imprinting Loss

Most mammalian autosomal genes are expressed on both the paternal and maternal autosomes. However, genetic imprinting means a few genes are expressed from only one parental chromosome. Epigenetic marks including DNA methylation, chromatin remodeling, and noncoding RNA regulation are critical for the maintenance of genetic imprinting [8]. The IGF-2 gene, the earliest found imprinted gene, is located at 11p15.5 and consists of 29.3 kb and 10 exons. Driven by five promoters, the IGF-2 gene encodes four IGF-2 precursors from a paternal chromosome [9]. The non-coding H19 gene, which is 128 kb downstream of the IGF-2 gene, is widely regarded as the IGF-2/H19 locus with the IGF-2 gene, because their genetic imprints are jointly determined by differentially methylated regions (DMRs) within the imprinting control region (ICR) [10]. The transcription regulator CCCTC-binding factor (CTCF), as a highly conserved zinc finger nuclear protein, inhibits gene expression by binding to different sites of gene promoters, enhancers, and insulators [11]. In the human genome, more than 15,000 CTCF binding sites have been identified [12], including the IGF-2/H19 locus. Under physiological conditions, DMRs on the paternal chromosome in the IGF-2/H19 locus are methylated, while on the maternal chromosome, they are not methylated [13,14]. The binding of CTCF to unmethylated ICR blocks downstream enhancer binding with IGF-2 promoter, thereby silencing IGF-2 gene expression on the maternal chromosome [14]. The loss of IGF-2 imprinting means the increased methylation of ICR, resulting in the increased expression of IGF-2. IGF-2 is expressed at the highest level in the embryonic period and is particularly important for regulating fetal growth and development [15,16]. Therefore, abnormal expression of IGF-2 in the embryonic period may lead to birth defects, such as the congenital disease Beckwith–Wiedemann syndrome (BWS) [17]. BWS, which is caused by increased expression of IGF-2, is mainly characterized by macroglossia, abdominal wall defects, overgrowth, an increased risk of neonatal hypoglycemia, and childhood neoplasms [18]. In addition, the loss of the gene imprinting of IGF-2 has also been observed in neoplasms, including hepatic carcinoma, breast carcinoma, lung cancer, cervical cancer, choriocarcinoma, etc., [19,20], which result from the increased expression of IGF-2 in tumors, promoting tumor cell growth. The increased methylation in ICR increases IGF-2 expression, whereas the loss of methylation on paternal chromosomes results in decreased IGF-2 expression. The low expression of IGF-2 may also lead to Russell–Silver syndrome (RSS), which is a growth deficiency syndrome characterized by prenatal growth defects, facial dysmorphic features, and developmental retardation [21]. In conclusion, the overexpression of IGF-2 may lead to severe growth and development disorders in both the embryonic period and the adult period and can even lead to tumor growth.

### 2.2. Binding of IGF-2 to Its Receptors

IGF-2 is a single-chain polypeptide that contains 67 amino acids and three disulfide bonds to hold together a specific three-dimensional structure [22]. At least 50% sequence homology and structural similarity exist between IGF-2 and the other ligands of the insulin family, including IGF-1 and insulin [23]. Both IGF-2 and IGF-1 are peptide hormones in circulation, and they exert significant functions in metabolic and developmental processes. IGF-2 mainly promotes proliferation and differentiation during pregnancy, while IGF-1 does so in puberty. IGF-2 is the most abundant IGF in adult circulation, with circulating concentrations three times higher than those of IGF-1 [3]. Although IGF-2 expression is low in adult tissues, small amounts of IGF-2 can be synthesized in normal skin dermal fibroblasts, lung fibroblasts, skeletal muscle cells, and adipocytes [24,25]. In addition, as a growth factor that promotes periodontal and fracture healing [26,27], IGF-2 is involved in the repair process of tissue injuries. During the development of skin wound healing, keratinocytes synthesize IGF-2 to promote tissue repair in an autocrine or paracrine manner [28,29]. The IGF-2 precursor encoded by the IGF-2 gene is a single-chain polypeptide consisting of an amino-terminal signal peptide (24 amino acids) and a carboxyl-terminal E domain (89 amino acids). Peptide cleavage, oxygen-linked glycosylation, and hydrolysis of the E domain culminate in the formation of mature IGF-2 [30,31,32].

There are three receptors that bind to IGF-2 and mediate the downstream signaling pathways. They are IGF-1R, insulin receptor (IR), and IGF-2R. IGF-1R and IR are structurally and functionally similar tyrosine kinase receptors, which are heterodimers consisting of two identical monomeric units. Each monomeric unit contains an extracellular α subunit and a transmembrane β subunit linked together by disulfide bonds [33]. During the synthesis of IR, the mRNA precursor transcribed from the gene is selectively spliced by exon 11 to form two isoforms, IR-A and IR-B, of which IR-A subtype does not express 12 amino acids coded by exons 11 [34]. In the state without binding to IGF-2, two monomers of IGF-2 receptors composed of α and β subunits present an inverted V-shaped conformation outside the cells. The transmembrane sites FnIII-3, one of the domains that make up the β subunits of the two monomers, are relatively far apart. After binding to their ligands, transmembrane sites FnIII-3 are close to each other, which makes the receptor J-shaped as a whole due to the changes to the receptor conformation, leading to the activation of intracellular tyrosine kinase [35,36].

IGF-2 binds to its receptors with different affinities. Because of its lower affinity for IR-B, IGF-2 exerts mitosis-promoting effects primarily through IGF-1R and IR-A with high affinity [37]. In addition, when the expression of IGF-1R is decreased, the binding of IR-A to IGF-2 can compensate for the loss of IGF-1R [38]. The binding affinity of the receptor to IGF-2 is consistent with the result that IGF-2 mainly acts through the signaling pathway mediated by IGF-1R and IR.

Due to the high sequence homologies between IGF-1R and IR, a monomeric structure composed of an α subunit and a β subunit can be randomly combined among different receptors to form new hybrid receptors, including IGF-1R:IR-A, IGF-1R:IR-B, and IR-A:IR-B [33]. Among these receptors, IGF-1R:IR-A and IGF-1R:IR-B heterodimers bind to ligands with a similar affinity for IGF-1R [39,40,41]. In conclusion, the two receptor forms of IGF-2 are flexible and varied, and different combinations can bind to IGF-2 and mediate downstream signaling pathways. Therefore, the changes in the downstream signaling pathways caused by IGF-2 are not restricted by its receptors but are more restricted by its own expression.

Unlike the above receptors, the IGF-2/cation-independent mannose-6-phosphate receptor (IGF-2R/CI-MPR) is a 300 kDa transmembrane glycoprotein that is not structurally or functionally similar to IGF-1R or IR. It is similar to a 46 kDa cation-dependent mannose-6-phosphate receptor (CD-MPR). Researchers generally consider that IGF-2R mainly transports lysosomal enzymes from the trans-Golgi network to endosomes and then to lysosomes. In the insulin family, IGF-2R has an extremely high affinity for IGF-2 and can target IGF-2 for lysosomal degradation to control IGF-2 levels in tissues and circulation [42]. In accordance with the result that IGF-2 promotes the growth and proliferation of tumor cells, IGF-2R is considered to be a tumor suppressor gene during cancer occurrence and development [43]. A recent study found that IGF-2 affects the inflammatory phenotype of macrophages through the regulation of proton channels in the cytoplasm. Low-dose IGF-2 tends to bind to IGF-2R and induce the internalization of IGF-2R in the nucleus. Then, the internalization of the IGF-2R promotes the entry of cytoplasmic H^+^ into the mitochondria through the GSK3α/β-Dnmt3a pathway and enhances the oxidative phosphorylation process of macrophages. Ultimately, the macrophages exhibit an anti-inflammatory phenotype. In contrast, large-dose IGF-2 primarily binds to IGF-1R and mediates GSK3α/β phosphorylation, thereby blocking proton channels and enabling macrophages to exhibit a pro-inflammatory phenotype [44]. In conclusion, IGF-2 may affect the occurrence and development of fibrosis by regulating the phenotype of macrophages.

IGF-2R can bind to a variety of ligands, such as M6P-containing molecules: leukemia suppressor, proliferator, and PXS64; and molecules not containing M6P: IGF-2, retinoic acid, TGF-β, and plasminogen [45,46]. IGF-2 and M6P bind with IGF-2R in different sites. M6P-containing ligands bind with IGF-2R at domains 3 and 9, while IGF-2 binds with IGF-2R at domain 11. We could not find any reports about the interactions between M6P-containing ligands and IGF-2 when they bind with IGF-2R, but we speculate that M6P-containing ligands and IGF-2 competitively bind with IGF-2R. Once one ligand binds with IGF-2R, this might result in the conformational change of IGF-2R, and the other cannot bind with IGF-2R. During a comparison of the binding kinetics of IGF-2R, no distinct differences were found between its affinity for IGF-2 and M6P. We speculate that which ligand binds with IGF-2R depends on the content of the ligand in the tissue microenvironment. It is known that the affinity of IGF-2 binding with IGF-2R is enhanced by the presence of fibronectin in domain 13 [47,48]. Fibronectin is necessary for many types of organs’ fibrosis progression, and the effect it has on IGF-2/IGF-2R may build a profibrotic microenvironment or a positive feedback loop of fibronectin/IGF-2/IGF-2R to promote organ fibrosis.

### 2.3. Downstream Signals of IGF-2

IGF-2 binding to IGF-1R/IR mainly triggers the intracellular phosphorylation cascade effect to transduce downstream signals through the classical mitogen-activated protein kinase (MAPK) and phosphatidylinositol-3-kinase/protein kinase B (PI3K/Akt) pathways [49]. The receptor’s intrinsic tyrosine kinase is activated when it is combined with IGF-2, leading to the autophosphorylation of tyrosine residues within the intracellular β-subunit, then recruits insulin receptor substrates (IRS) 1–4, further binds to and activates PI3K and Akt, and ultimately activates downstream signaling molecules, such as Bad, which can inhibit cell apoptosis. In addition, the receptor’s intrinsic tyrosine kinase also activates the mammalian target of rapamycin (mTOR), further promoting ribosomal protein synthesis for cell mitosis [50]. Additionally, the receptor’s intrinsic tyrosine kinase activates ERK in the Ras/Raf/MEK/ERK pathway signaling, which can directly enter into the cell nucleus to act on transcription factors, such as c-fos or c-jun, and then promote a mitotic response to participate in cell proliferation, differentiation, and apoptosis [51].

Although IGF-2 performs its functions mainly via the two abovementioned signaling pathways, IGF-2 exerts different effects in different cell types under physiological and pathological conditions, which may be related to an unequal binding affinity between the ligand and its receptors or distinct activated downstream substrates [52]. In cancer research, researchers have found that IGF-2 is overexpressed in many cancer types, as well as its receptors. Excessive IGF-2 could promote tumorigenesis and progression and result in undesirable chemotherapy prognosis [53]. For example, in ovarian cancer, tumor growth and angiogenesis could be inhibited while cell apoptosis could be enhanced by RNA interference with IGF-2’s receptor [54]. A monoclonal antibody against IGF-1R, AMG-479, and its kinase inhibitor, MK0406, effectively inhibit the proliferation of ovarian tumor cells and enhance tumor sensitivity to chemotherapeutic drugs such as cisplatin and taxane, which indicates that IGF-2 is an effective target for cancer therapy [55]. Moreover, a small molecule inhibitor of IGF-2, chromeceptin, can inhibit IGF-2 expression in 3T3, HepG2 cell lines, and human hepatocellular primary cells. Chromeceptin can reduce the vitality of hepatocellular primary cells and inhibit the proliferation and migration of 3T3 cell lines [56,57]. In addition, IR-A is also overexpressed in various neoplasms (thyroid cancer, breast cancer, prostate cancer, and colorectal cancer) [58,59,60]. Autocrine or paracrine loops of overexpressed IGF-2/IR-A are established in thyroid cancer and breast cancer cells [61,62]. IGF-2 and its receptors are also overexpressed in various stem cells and further promote the proliferation and self-renewal of many types of stem cells. For example, IR-A and IGF-1R are abundantly expressed in the neural stem cells, and heterodimeric receptors formed by IR-A can maintain the renewal of tumor stem cells or neural stem cells [63,64]. By knocking out IR or IGF-1R, it was found that IGF-2 promotes neural progenitor cell proliferation through IGF-1R and is crucial for neural stem cell renewal through IR [65]. In addition, the IGF-2/ERK signaling pathway enhances hepatic stem cell proliferation and self-renewal in patients with portal hypertension [66], and specific knockout of IGF-2 results in intestinal stem cell growth inhibition [39]. Furthermore, IGF-2 produced by fibroblasts regulates human embryonic stem cells’ self-renewal and survival through IGF-1R [67].

The IGF-2-IGF-1R/IR signaling pathway is complex. IGF-1R can heterodimerize or trimerize with other tyrosine kinase receptors, such as EGFR, HER2, and HER3 [68], and mediate the MAPK or PI3K/Akt signaling pathway. Therefore, when IGF-1R expression is decreased, other receptor tyrosine kinases will compensate for the loss of downstream signaling following IGF-1R inhibition, causing the overexpression of HER2 in ovarian cancer cells and promoting both cell proliferation and differentiation to benefit the development of cancer as far as possible [69].

## 3. IGF-2 and Fibrosis

### 3.1. Skin Fibrosis

Skin wound healing consists of three stages: inflammation, proliferation, and remodeling [70]. Epidermal damage can be repaired by the extension of surrounding epidermal cells, usually without significant scar formation. However, when severe injuries, such as burns, cause damage to the skin dermis layer, proliferative fibroblasts secrete large amounts of extracellular matrix, resulting in an imbalance between collagen synthesis and breakdown and eventually leading to hypertrophic scar and keloid formation [71]. The changes in the various components of the extracellular matrix cause the stiffness and height of hypertrophic scars to differ significantly from those of the surrounding normal skin. The aesthetic impact and functional limitations caused by scar contractures usually cause great obstacles to patients, leading to symptoms such as pain, itching, and depression.

Several researchers have investigated the expression of IGF-2 in skin fibrosis. Yanghong Hu et al. found that the expression of the IGF-2 gene is significantly upregulated in hypertrophic and keloid scars through bioinformatics [5]. Similarly, Zhenfu Hu et al. found that IGF-2 gene expression in hypertrophic and keloid scars was 5.67 and 27.87 times that of normal skin, respectively, using gene expression chips. In addition, other insulin family growth factors, such as IGF-1 and insulin, are not significantly elevated in hypertrophic and keloid scars compared to normal skin [72]. This suggests that IGF-2 is more likely to be involved in skin fibrosis than other ligands. Moreover, as a recognized organ fibrosis-promoting factor, TGF-β1 can promote collagen deposition in wound tissues [73]. IGF-2 can promote TGF-β1 expression and secretion in dermal fibroblasts, suggesting that IGF-2 plays a promoting role in hypertrophic scar formation [74].

Rolfe KJ et al. also found that dermal fibroblasts respond differently to IGF-2 in the prenatal and postnatal periods. Fetal skin wounds have good healing capabilities without scar formation. IGF-2-stimulated fetal dermal fibroblasts show only an unidentified tyrosine-phosphorylated protein overexpression without significant collagen synthesis. Fetal fibroblasts are much less responsive to IGF-2-mediated mitosis than postnatal dermal fibroblasts, which may be related to the transition from scarless to scarred wound healing [75].

### 3.2. Pulmonary Fibrosis

Systemic sclerosis (SSc)-related pulmonary fibrosis and idiopathic pulmonary fibrosis (IPF) are both chronic lung diseases, characterized by interstitial fibrosis, that lead to a progressive decline in pulmonary function [76]. Primary SSc lung fibroblasts show a four-fold increase in IGF-2 mRNA expression and a two-fold increase in IGF-2 protein expression compared to normal lung fibroblasts. IGF-2 expressed in SSc lung fibroblasts induces collagen type I and fibronectin overexpression in a dose- and time-dependent manner via the activation of PI3K and JNK [77]. JNK can enter the nucleus and activate a variety of downstream transcription factors, such as c-jun, c-fos, and ATF-2. The transcription factor complex, activator protein-1 (AP-1), formed by c-jun and c-fos promotes the expression of collagen type I by binding to its promoter. Similarly, the heterodimer formed by c-jun and ATF-2 promotes its transcription by binding to the promoter of fibronectin [78,79,80]. Additionally, IGF-2 is significantly increased in the lung fibroblasts of IPF patients and promotes collagen and fibronectin deposition, and the blocking of endogenous IGF-2 or IGF-1R/IR expression inhibits extracellular matrix production.

However, it is debated whether IGF-2 can promote lung fibroblast proliferation and fibrotic proteins synthesis. Hetzel’s early research indicated that IGF-2 stimulated normal lung fibroblast proliferation but had no pro-proliferative effect on IPF lung fibroblasts [81]. Additionally, Eileen Hsu et al. applied different concentration gradients of IGF-2 to stimulate normal and SSc pulmonary fibroblasts. However, the results showed that IGF-2 had no effect on the proliferation of these fibroblasts [77]. It is noteworthy that these differences may be attributable to different IGF-2 concentrations and stimulation times. Eileen Hsu et al. set the IGF-2 concentration at 0, 50, 100, 150, and 200 ng/mL and stimulated lung fibroblasts for 48 h, whereas Hetzel selected a concentration of 30 ng/mL and stimulated fibroblasts for 36 h. This suggests that the effective concentration of IGF-2 for promoting lung fibroblast proliferation may be limited to lower concentrations and that its pro-proliferative effect may not be concentration-dependent or time-dependent. In addition, the role of IGF-2 in promoting collagen and fibronectin production by lung fibroblasts is also debatable. Eileen Hsu showed that IGF-2 stimulated collagen and fibronectin synthesis in SSc and normal lung fibroblasts and the stimulation effect of IGF-2 on SSc lung fibroblasts was more obvious than on normal lung fibroblasts. SSc lung fibroblasts may be more sensitive to IGF-2 compared to normal lung fibroblasts. Hetzel also found that IGF-2 promoted fibronectin synthesis in IPF lung fibroblasts. However, in normal lung fibroblasts, IGF-2 stimulation reduced fibronectin synthesis. The researchers do not elaborate further on these results, which may be related to the differences in the cell lines used.

IGF-2 also promotes pulmonary fibrosis through other mechanisms. For example, IGF-2 increases the expression of TGF-β2 and TGF-β3 and activates downstream Smad 2/3 signaling. Interestingly, TGF-β1 receptor inhibitors block the role of IGF-2 in differentiating fibroblasts into myofibroblasts, indicating that there is a close relationship between IGF-2 and TGF-β1 [4]. In addition, the matrix metalloproteinase (MMP) family can mediate the breakdown of the extracellular matrix, so the tissue inhibitor of MMP (TIMP) can promote extracellular matrix deposition [82] while IGF-2 also breaks the TIMP:MMP balance and promotes an intracellular and extracellular fibrotic environment [83].

### 3.3. Myocardial Fibrosis

IGF-2 and its receptors play an important role in normal cardiac morphogenesis and growth in embryos. IGF-2R-deficient mice have enlarged hearts and abnormal development of the interventricular septum [84]. In addition, through gene microarray studies of differential genes in fetal and adult myocardia, Zhimin Geng identified IGF-1R as a key gene that regulates cardiomyocyte proliferation. Fetal epicardium synthesizes and releases IGF-2 and acts as a major mitogenic factor, promoting cardiomyocyte proliferation by binding to IGF-1R and IR-A. The circulating IGF-2 of newborns can promote cardiomyocyte proliferation and hypertrophy via the excessive activation of the mTOR signaling pathway [85].

Cardiomyocytes remodel the extracellular matrix under abnormal pathological conditions, such as hypertension and myocardial infarction [86]. Researchers have identified cardiomyocytes in pathological states that overexpress IGF-2, such that IGF-2 and IGF-2R expression are significantly upregulated in hypertensive rats treated with abdominal aortic ligation and in angiotensin II (Ang II)-treated cardiomyocytes [87]. Ang II promotes IGF-2 expression in cardiomyocytes via the ERK and JNK pathways. This effect can be blocked by U-0126, a MEK inhibitor, and SP-600125, a JNK inhibitor [86,88]. Moreover, the increased expression of IGF-2 and IGF-2R was also observed in myocardial scar tissue. IGF-2 analog [Leu27]IGF-2 can specifically activate IGF-2R, and the transfection of [Leu27]IGF-2 into rat cardiomyocytes by adenovirus can induce reconstruction characterized by pathological hypertrophy, myocardial fibrosis, and cardiomyocytes’ apoptosis accompanied by massive collagen deposition [89]. In an IGF-2-overexpressed imprinted mouse model, IGF-2 induced cardiomyocyte hypertrophy through downstream Akt and mTOR signaling after binding to IGF-1R and could be inhibited by an IGF-1R inhibitor (BMS754807) and an mTOR inhibitor (rapamycin) [90]. In addition to rodents, patients with BWS characterized by IGF-2 overexpression also develop cardiomyopathy characterized by myocardial hypertrophy and myocardial fibrosis as pathological features [85].

Compared to IGF-1R, IGF-2R is a more complex receptor that binds not only to IGF-2 but also to other M6P-containing ligands to regulate different cellular functions [91]. Previously, IGF-2R was thought to function as a clearance receptor for IGF-2. For example, increased IGF-2 levels due to the disruption of IGF-2R could promote cardiomyocyte proliferation and reduce hypoxia-induced apoptosis in rat cardiomyocytes. In addition, it has also been shown that IGF-2 induces cardiomyocyte hypertrophy and apoptosis via IGF-2R. In H9c2 cardiomyocytes, IGF-2 binding to IGF-2R stimulates the downstream Gaq protein to phosphorylate PLC-β, thereby activating caspase 3 to induce apoptosis in cardiomyocytes [92]. IGF-2/IGF-2R can also increase plasminogen activator (PA) expression to promote the occurrence of myocardial fibrosis through disrupting the balance of MMP-9/TIMP-2 expression [93]. In addition, IGF-2/IGF-2R induces pathological cardiac hypertrophy through the calcium/calmodulin-dependent protein kinase II (CaMKIIδ) and calcineurin signaling pathways, which can be alleviated by combining inhibitors of CaMKIIδ and calcineurin [87,93]. It has been confirmed by earlier studies that IGF-2R can also result in extracellular matrix remodeling via the activation of TGF-β and plasminogen through potential proteolysis and cleavage after binding to TGF-β and plasminogen but not IGF-2 [94].

### 3.4. Liver Fibrosis

Liver fibrosis is the final stage of various chronic liver diseases (viral hepatitis and drug or alcohol-induced or metabolic liver disease). As liver damage increases, the liver parenchyma is continuously replaced by scar tissue [95]. Hepatic stellate cells (HSCs) are the main cell type in the liver that promotes hepatic fibrosis. They are quiescent in physiological states, while injured hepatocytes differentiate into highly proliferative myofibroblasts and synthesize a large amount of collagen and fibronectin under pathological conditions [96].

In both a carbon tetrachloride (CCl_4_)-induced mouse model of liver fibrosis and a 2-fluorenylacetamide (2-FAA)-induced rat model of liver fibrosis, hepatocytes overexpressed IGF-2 [97,98]. Javier Vaquero shows that IGF-2 can promote LX2 cell (human hepatic astrocyte lines) proliferation via IGF-1R/IR, activate their conversion to myofibroblasts, and promote collagen and α-SMA expression. This effect can be inhibited by linstinib, an IGF-1R inhibitor [99]. In addition, hepatocellular carcinoma-associated fibroblasts promote α-SMA synthesis and enhance their contractility through IGF-1R and autocrine IGF-2 [100]. NK cells with a high killing effect can kill HSCs and thus inhibit liver fibrosis, and some investigators have suggested that IGF-2 may promote the killing effect of NK cells through the START3 signaling factor [101]. This is in contradiction to the abovementioned role of IGF-2 in promoting liver fibrosis. At present, little is known about the relationship between IGF-2 and NK cells. We speculate that fibrotic hepatocytes may inhibit the progression of fibrosis through the interaction between IGF-2 and NK cells, which may be a defense mechanism.

ROCK (serine/threonine protein (Rho) kinase) can promote HSC activation and myofibroblast migration, and its inhibitor, Y27632, can reduce the degree of fibrosis in animal models of liver fibrosis [102]. IGF-2R is significantly overexpressed in activated HSCs. More importantly, M6PHSA, as a synthetic compound, can be taken up by cells through binding to IGF-2R with a high affinity. Therefore, it has been processed to carry anti-fibrotic drugs in vitro. In in vitro experiments, compounds constructed from M6PHSA and Y27632 could be specifically taken up by activated hepatic stellate cells and reduce the expression of fibrotic markers such as α-SMA. Furthermore, coupled injections of Y27632 and M6PHSA into a carbon tetrachloride-induced liver fibrosis model in mice inhibited liver fibrosis [103]. Similarly, several other drugs with anti-proliferative or anti-fibrotic potential, such as doxorubicin, mycophenolic acid, LY-364947, and ALK5 (an inhibitor of TGF-β1 receptors) can also be combined with M6PHSA to exert anti-fibrotic effects [104,105]. Interestingly, other studies found that in addition to HSCs, Kupffer cells and endothelial cells in fibrotic livers also efficiently take up M6PHSA. However, polyinosinic acid can competitively inhibit scavenger receptors other than IGF-2R, inhibiting Kupffer cells and endothelial cells while enhancing HSC-specific uptake of anti-fibrotic drugs carried by M6PHSA [106].

Circulating IGF-2 is mainly produced by hepatocytes [107], and IGF-2 secreted by injured hepatocytes can promote hepatocyte proliferation. IGF-2 enrichment can be observed in proliferating hepatocytes from patients with liver fibrosis [97]. In addition, studies have found that miR-615-5p, which downregulates IGF-2 mRNA, is highly expressed in liver cirrhosis and hepatocellular carcinoma tissues, and miR-615-5p targets IGF-2 to inhibit the proliferation of hepatocytes [108]. In addition, IGF-2 has been identified as a potential biomarker in liver diseases. For example, plasma IGF-2 and EGFR can be used as diagnostic markers for liver fibrosis in obese patients [6]. Specific hypomethylation of the IGF-2 gene in hepatocytes and peripheral blood mononuclear cells is a risk predictor of hepatic carcinoma in patients with hepatitis C cirrhosis [109,110].

### 3.5. Renal Fibrosis

Renal fibrosis is characterized by renal tubular cell apoptosis, tubulointerstitial fibroblast proliferation, extracellular matrix (ECM) deposition, and inflammatory cell infiltration. It is a common result of multiple chronic kidney diseases (CKD) and can ultimately lead to renal failure and even death [111]. miR-483 is a conserved sequence encoded by the intron region of the IGF-2 gene. After processing, two mature miRs, miR-483-3P and miR-483-5P, can be generated. miR-483-5P promotes the transcription of the IGF-2 gene by binding to the promoter expressed in the fetus period [112]. Their co-overexpression in various cancers has been confirmed, and their co-expression in rectal cancer makes miR-483-5P a possible molecular chaperone of IGF-2 [113]. As a key factor in fetal growth and development, IGF-2 is expressed in immature tubular epithelial cells, mature glomerular epithelial cells, and fibroblasts (interstitial cells) in the renal medulla, which is involved in the fetal kidney developmental process [114]. In a model of renal fibrosis induced by unilateral ureteral obstruction, it was found that the expression of miR-483, IGF-2, and the pro-fibrotic factors TGF-β and CTGF were increased, and the anti-fibrotic factor bone morphogenetic protein (BMP7) was decreased The above factors were confirmed by Pearson correlation analysis to be correlated with miR-483 [115]. In addition to the above factors, the target gene prediction of miR-483 in this study also involved a variety of cytokines, such as PDGF, TIMP2, BMP1, etc., suggesting that they may directly or indirectly participate in renal fibrosis through various pathways.

### 3.6. Other Fibrosis

Dupuytren’s disease is an autosomal dominant fibroproliferative disease that is characterized by palmar fascia fibrosis with permanent digital contractures. The overexpressed IGF-2 in contracture palmar fascia tissue significantly enhanced cell contractility, and this effect could be abolished by IGFBP-6, suggesting that IGFBP-6 and IGF-2 may be the regulators of the proliferation and contractility of the cells in contracture tissue [116]. Similar to Dupuytren’s disease, frozen shoulder syndrome also shows elevated expression of IGF-2. It is a connective tissue disease characterized by the fibrosis of the shoulder joint capsule [117]. In addition, betel nuts promote the occurrence of oral submucosal fibrosis (OSF) by inducing IGF-2 expression in gingival fibroblasts [118]. IGF-2R was observed to promote the assembly of α-SMA into fibers in damaged keratocytes and to promote their differentiation into myofibroblasts [42].

## 4. Discussion and Conclusions

IGF-2, as a pro-proliferative and pro-fibrotic factor, is generally elevated in fibrotic diseases. As ligands in the insulin family, IGF-2 and IGF-1 have similarities in terms of their structures and functions. We infer that IGF-2-mediated fibrosis can be superior to IGF-1-mediated fibrosis for the following reasons. First, in certain fibrotic tissues, such as hypertrophic and keloid scars, IGF-2 is significantly elevated compared to normal tissues while IGF-1 does not change significantly. Second, IGF-2 is the most abundant IGF in circulation compared to IGF-1. Third, both IGF-2 and IGF-1 function by binding to IGF-1R and IR, but IGF2 can also induce pathological cardiac fibrosis through calcium/calmodulin-dependent protein kinase II (CaMKIIδ), the calcineurin signaling pathway, and disrupting the balance of MMP-9/TIMP-2 expression.

**Figure 1:**
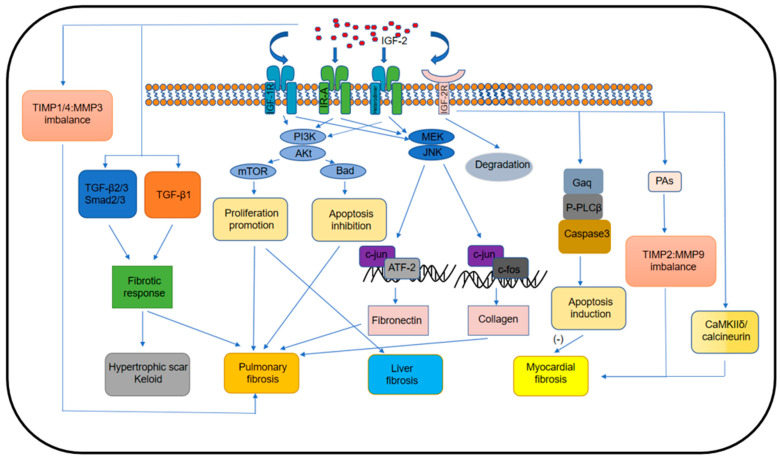
Molecular pathways of IGF-2 promotes organ fibrosis. By binding to IGF-1R, IR, and their heterodimers, IGF-2 actives the PI3K/Akt and JNK/c-jun signaling pathways to promote fibroblast proliferation, inhibit their apoptosis, and increase extracellular matrix (fibronectin and collagen) synthesis. IGF2 can also promote organ fibrosis by disrupting the TIMP/MMP balance and activating CaMKIIδ and calcineurin, as well as TGF-β signaling.

As is shown in Figure 1, the regulation mechanism of IGF-2 and its receptors for the fibrosis process in different organs is relatively complex. Table 1 summarizes that inhibitors of IGF-2 and its receptors downregulate IGF-2-stimulated extracellular matrix production. The fibrotic models used in this review are summarized in Table 2 and Table 3. The most commonly used animal models are rodents. IGF-2 exacerbates the progression of fibrosis I n different models of organ fibrosis. At present, some known mechanisms of IGF-2 upregulation in fibrotic tissues have been revealed. First, hypoxia induces the overexpression of IGF-2 in several gastric cancer cell lines and HepG2 hepatocellular carcinoma cell lines [119,120]. Additionally, it is widely reported hypoxia can promote fibrosis via the promotion of fibroblast proliferation, collagen synthesis, and apoptosis inhibition [121,122]. In addition, miR-4521 targets the 3′UTR region of IGF-2 to silence IGF-2 expression in gastric cancer cells, whereas hypoxia can promote IGF-2 expression by downregulating miR-4521 [120]. Second, in an Ang II-stimulated mouse model of myocardial fibrosis, Ang II promotes IGF-2 expression in cardiomyocytes via the ERK pathway and JNK pathway. This effect could be blocked by U-0126, an MEK inhibitor, and SP-60012, a JNK inhibitor [86,88]. Third, patients with BWS characterized by myocardial fibrosis have increased IGF-2 expression. The mechanism is due to a loss of IGF-2 gene imprinting [18]. Fourth, cardiomyocytes derived from patients with heart failure overexpress IGF-2 compared to normal cardiomyocytes, creating a pro-fibrotic microenvironment, which continues to promote IGF-2 production. Although the mechanism of IGF-2 overexpression in cardiomyocytes from heart failure at the onset is not known, the formation of an autocrine fibrotic ring plays a role in maintaining high levels of IGF-2 and a fibrotic environment [123]. Finally, our unpublished results found that mechanical tension induced IGF-2 overexpression in skin cells. The specific molecular mechanisms involved are under investigation.

It is thought that IGF-2-mediated organ fibrosis in adult humans and rodents has similar mechanisms. For example, IGF-2 promotes the proliferation of adult lung fibroblasts through the PI3K/Akt/mTOR signaling pathway. Meanwhile, in an IGF-2-overexpressed genetic imprinted mouse model, IGF-2 also promoted cardiomyocyte proliferation and fibrosis via the Akt/mTOR signaling pathway [77,90]. Although the pathway in which IGF-2 mediates organ fibrosis in both species has similarities, the circulating IGF-2 level in adult humans and rodents is different. Although the IGF-2 gene is imprinted in both adult humans and rodents, the promoters of the IGF-2 gene are not identical between the two species [124]. In adult humans, circulating IGF-2 is mainly expressed in hepatocytes by double alleles via promoter P1, thus maintaining circulating IGF-2 at a high concentration [107]. In contrast, rodents lack promoter P1, so their circulating IGF-2 concentrations decline dramatically after birth. Adult rodents have virtually no circulating IGF-2 [125]. Adult rodents’ tissues also have very low levels of IGF-2 expression. IGF-2 expression in the nervous system of adult rodents is high [124]. Compared to rodents, adult humans have higher levels of IGF-2 in their circulation. Thus, the degree of organ fibrosis in humans is perhaps heavier than in rodents with the same organ fibrosis and pathological features.

Overall, IGF-2 and its receptors can activate the known TGF-β pro-fibrotic pathway and disrupt the balance of matrix proteases and their inhibitors, leading to excessive ECM deposition and inducing the differentiation of fibroblasts into myofibroblasts, thus promoting the occurrence and development of fibrosis. However, in hypertrophic scars, oral submucosal fibrosis, hereditary palmar fascia contractures, and renal fibrosis, there are few studies on IGF-2’s effect on fibrosis, and current studies mainly focus on the differences in IGF-2 expression. The expression changes and mechanisms of IGF-2 during the development of fibrotic disease and wound healing are still worth exploring.

**Table 3:** Verified IGF-2-overexpressed animal organ fibrosis models.

Organ	Fibrotic Model	Sample Source
Lung	Bleomycin-induced pulmonary fibrosis via trachea injection [126]	Mice
Heart	Hypoglycemia-induced right ventricular fibrosis [93]	Sheep
	Myocardial fibrosis in an imprinted mouse model [90]	Mice
Liver	CCl_4_-induced liver fibrosis [97]	Mice
	2-FAA-induced liver fibrosis [98]	Rat
Kidney	Renal fibrosis induced by unilateral ureteral obstruction [115]	Mice

## Figures and Tables

**Table 1:** Inhibitors of IGF-2-stimulated ECM production.

Reagent	Type	Cells	ECM Production with IGF-2 Stimulation	Reference
LY294002	PI3K inhibitor	SSc and normal lung fibroblasts	Downregulated	[77]
Jnk II inhibitor	JNK inhibitor	SSc and normal lung fibroblasts		
Tyrphostin AG 538	IGF-1R tyrosine kinase inhibitor	SSc and normal lung fibroblasts	Downregulated	
αIGF-2	Neutralizing antibody of IGF-2	SSc, IPF and normal lung fibroblasts	Downregulated	[4]
αIGF-1R	Neutralizing antibody of IGF-1R	SSc, IPF and normal lung fibroblasts	Downregulated	
αIR	Neutralizing antibody of IR	SSc, IPF and normal lung fibroblasts	Downregulated	
ALK5	TGF-β1 receptor inhibitor	Hepatic stellate cells	Downregulated	[102,103,104,105]
Y27632	Serine/threonine protein (Rho) kinase inhibitor	Hepatic stellate cells	Downregulated	

**Table 2:** Verified IGF-2-overexpressed human organ fibrosis models.

Organ	Fibrotic Model	Sample Source
Skin	Burns and laser-induced keloid scars [5]	Human
	Hypertrophic scar from burns and postpartum [72]	
Lung	Systemic sclerosis induced by autoimmune inflammatory response [77]	
	Idiopathic pulmonary fibrosis induced by genetic, autoimmune inflammatory response, viral infection, or drugs [4]	
Heart	Myocardial fibrosis in BWS patients [85]	
Liver	Hepatitis C virus infection-induced liver fibrosis [109,116]	
Palm	Palmar fascia fibrosis in autosomal dominant inheritance [116]	
Shoulder	Degenerative shoulder capsule fibrosis [117]	
Oral cavity	Areca nut extract-induced oral submucosal fibrosis [118]	

## Abbreviations

IGF-1	Insulin-like growth factor 1
IGF-2	Insulin-like growth factor 2
ECM	Extracellular matrix
IR	Insulin receptor
IGF-1R	Insulin-like growth factor receptor 1
IGF-2R	Insulin-like growth factor receptor 2
DMRs	Differentially methylated regions
ICR	Imprinting control region
CTCF	CCCTC-binding factor
BWS	Beckwith–Wiedemann Syndrome
RSS	Russell–Silver syndrome
CI-MPR	Cation-independent mannose-6-phosphate receptor
CD-MPR	Cation-dependent mannose-6-phosphate receptor
GSK3	Glycogen synthase kinase 3
MAPK	Mitogen-activated protein kinase
PI3K	Phosphatidylinositol-3-kinase
Akt	Protein kinase B
IRS	Insulin receptor substrates
mTOR	Mammalian target of rapamycin
ERK	Extracellular-regulated protein kinase
c-fos	Fos proto-oncogene, AP-1 transcription factor subunit
c-jun	Jun proto-oncogene, AP-1 transcription factor subunit
EGFR	Epidermal growth factor receptor
HER2 (ERB2)	Erb-b2 receptor tyrosine kinase 2
HER (ERBB3)	Erb-b2 receptor tyrosine kinase 3
SSc	Systemic sclerosis
IPF	Idiopathic pulmonary fibrosis
JNK (MAPK8)	Mitogen-activated protein kinase 8
AP-1	Activator protein 1
ATF-2	Activating transcription factor 2
MMP	Matrix metalloproteinase
Ang II	Angiotensin II
TIMP	Tissue inhibitor of metalloprotease
Gaq (GNAQ)	G protein subunit alpha q
PLC-β	Phospholipase C beta
PA	Plasminogen activator
CaMKIIδ	Calcium/calmodulin-dependent protein kinase II
HSC	Hepatic stellate cell
CCl_4_	Carbon tetrachloride
2-FAA	2-fluorenylacetamide
α-SMA	α-smooth muscle actin
CKD	Chronic kidney disease
CTGF	Connective tissue growth factor
BMP	Bone morphogenetic protein
PDGF	Platelet-derived growth factor
OSF	Oral submucosal fibrosis
